# Development and testing of indicators to measure coordination of clinical information and management across levels of care

**DOI:** 10.1186/s12913-015-0968-z

**Published:** 2015-08-13

**Authors:** Marta-Beatriz Aller, Ingrid Vargas, Jordi Coderch, Sebastià Calero, Francesc Cots, Mercè Abizanda, Joan Farré, Josep Ramon Llopart, Lluís Colomés, María Luisa Vázquez

**Affiliations:** Health Policy and Health Services Research Group, Health Policy Research Unit, Consortium for Health Care and Social Services of Catalonia, Avenida Tibidabo, 21, 08022 Barcelona, Spain; Grup de Recerca en Serveis Sanitaris i Resultats en Salut, Serveis de Salut Integrats Baix Empordà, Carrer Hospital, 17-19 Edif. Fleming, 17230 Palamós, Spain; Catalan Health Institute, Gran Via de les Corts Catalanes, 587, 08007 Barcelona, Spain; IMIM - Hospital del Mar Medical Research Institute, Carrer Dr. Aiguader, 88, 08003 Barcelona, Spain; Institut de Prestacions d’Assistència Mèdica al Personal Municipal, Carrer Viladomat, 127, 08015 Barcelona, Spain; Centre Integral de Salut Cotxers, Avinguda de Borbó, 18 - 30, 08016 Barcelona, Spain; Health Policy and Health Services Research Group; Division of Management, Planning and Organizational Development, Badalona Healthcare Services, Via Augusta, 9-13, 08911 Badalona, Spain; Health Policy and Health Services Research Group; Strategic Planning Division, SAGESSA Group, Avinguda del Dr. Josep Laporte, 2, 43204 Reus, Spain

**Keywords:** Quality indicators, Coordination across levels of care, Clinical management coordination, Clinical information coordination, Health services research

## Abstract

**Background:**

Coordination across levels of care is becoming increasingly important due to rapid advances in technology, high specialisation and changes in the organization of healthcare services; to date, however, the development of indicators to evaluate coordination has been limited. The aim of this study is to develop and test a set of indicators to comprehensively evaluate clinical coordination across levels of care.

**Methods:**

A systematic review of literature was conducted to identify indicators of clinical coordination across levels of care. These indicators were analysed to identify attributes of coordination and classified accordingly. They were then discussed within an expert team and adapted or newly developed, and their relevance, scientific soundness and feasibility were examined. The indicators were tested in three healthcare areas of the Catalan health system.

**Results:**

52 indicators were identified addressing 11 attributes of clinical coordination across levels of care. The final set consisted of 21 output indicators. Clinical information transfer is evaluated based on information flow (4) and the adequacy of shared information (3). Clinical management coordination indicators evaluate care coherence through diagnostic testing (2) and medication (1), provision of care at the most appropriate level (2), completion of diagnostic process (1), follow-up after hospital discharge (4) and accessibility across levels of care (4). The application of indicators showed differences in the degree of clinical coordination depending on the attribute and area.

**Conclusion:**

A set of rigorous and scientifically sound measures of clinical coordination across levels of care were developed based on a literature review and discussion with experts. This set of indicators comprehensively address the different attributes of clinical coordination in main transitions across levels of care. It could be employed to identify areas in which health services can be improved, as well as to measure the effect of efforts to improve clinical coordination in healthcare organizations.

## Background

Healthcare systems are in a constant process of adaptation due to rapid advances in technology, new treatments, high specialisation and changes in the organization of health services [1]. As a consequence, patients are seen by an ever-expanding array of different providers in a variety of locations, making coordination difficult [1, 2]. This is particularly challenging in the care of patients with chronic and multiple conditions, who tend to use healthcare services more frequently and use a greater array of services than other patients [3, 4]. Clinical coordination across levels of care should prevent wasteful duplication of diagnostic testing, perilous polypharmacy and conflicting care plans [5, 6]; thus the effects of clinical coordination extend beyond cost reduction through improving quality of care [7–9].

This study is set within a conceptual framework for analysing the performance of integrated healthcare networks, which is based on an extensive literature review [6, 10] and could be applied in any healthcare area that arranges to provide a coordinated continuum of services to a defined population. In this framework, clinical coordination, together with continuity of care and access to health services, is considered an intermediate objective of integrated healthcare networks and is regarded as a means by which to reach the ultimate objectives of quality of care, efficiency and equity of access [6, 10, 11]. To analyse the achievement of these objectives, both external and internal processes and contextual factors are taken into account, as well as the different perspectives (services, professionals and users) and approaches.

In this conceptual framework, clinical coordination is defined as the harmonious connection of the different health services needed to provide care to a patient throughout the care continuum in order to achieve a common objective without conflicts [10, 12]. Continuity of care refers to how individual patients experience coordination of services, and it is defined as the degree to which patients experience care over time as coherent and linked [1]. Clinical coordination across levels of care consists of the coordination of both clinical information and clinical management [6, 10]. Clinical information coordination is the transfer and use of patients’ clinical information in order to harmonize activities between providers, and consists of two dimensions: transfer of clinical information and the use of this information [13]. Clinical management coordination is the provision of care in a sequential and complementary way according to a healthcare plan shared by the different services and healthcare levels involved, and consists of three dimensions: care coherence (i.e., the existence of similar approaches and treatment objectives among professionals from different levels of care), follow-up across care levels (i.e., the adequate monitoring of the patient when there are transitions from one care setting to another) and accessibility across levels (provision of care without interruption across levels of care throughout the clinical episode of the patient) [13].

The results of clinical coordination can be assessed by analysing processes aimed at coordination or their outputs (immediate results of activities related to clinical coordination) or outcomes (final expected middle-long term results of clinical coordination, such as hospital readmissions or avoidable hospital admissions), and using different perspectives (services, professionals, users (continuity)). The focus of this study relies on measures to assess the outputs of clinical coordination across levels of care (primary and secondary) by using service-based indicators.

Despite the interest this subject has generated, there are still important gaps in terms of measures to assess clinical coordination across levels of care and the development of new indicators continues to be considered a priority in health policy and health services research [14, 15]. Many of the attempts to address this to date have focused on developing indicators to measure healthcare outcomes which are attributed to improvements in clinical coordination [16]. However, the development of output indicators has been limited, and without this type of indicators it is not possible to conclude that outcomes in health care can be attributed to improvements in clinical coordination across levels of care [15].

Existing sets of indicators are usually designed to analyse a single dimension (e.g. transfer of information) or attribute (e.g. due completion of referral forms and discharge reports) of clinical coordination [17–20]. Those which address more than one dimension of clinical coordination are not exhaustive in their approach to clinical coordination and are often insufficiently operative or are not directed at the assessment of clinical coordination across levels of care [21–25]. Furthermore, the conceptual framework used to develop these measures is not generally explained in detail, so it is not obvious exactly which aspects of clinical coordination are being analysed or how measures relate to clinical coordination.

As a result of these issues, there is an overrepresentation of some dimensions of clinical coordination addressed by indicators, whilst other dimensions have scarcely been investigated [26]. Studies have concentrated in particular on the transfer of clinical information [22–24, 27–30], especially in terms of completeness of information in discharge reports [22, 30–34] and to a lesser degree in emergency reports [30] and referral forms [20, 35], and on the follow-up of patients and accessibility across care levels [22, 24, 29, 30, 36]. Only a few studies have used indicators to measure clinical coherence between care levels [30, 37].

The aim of this study is to develop and test a set of output indicators to comprehensively evaluate clinical coordination across care levels of care, i.e. addressing both types of clinical coordination, information and management, and their dimensions and attributes.

## Methods

The study consisted of two phases: in the first phase, a set of indicators to measure clinical coordination across levels of care was developed based on the literature review and expert discussions, and in the second phase, the set was tested in three different healthcare areas.

### 1. Development of a set of indicators to measure clinical coordination across levels of care

#### Identification of indicators: literature review

The study was based on the conceptual framework for analysing the performance of integrated healthcare networks [6, 10], which identifies two types of clinical coordination across levels of care (clinical information and clinical management) and five dimensions (transfer of information, use of information, care coherence, follow-up across levels and accessibility across levels). A systematic review of literature was undertaken to identify previously developed indicators. A computerised search of the following bibliographic databases was conducted: Pubmed, Social Science Citation Index, Science Citation Index, ECONLIT, CINAHL and LILACS, in addition to standard internet search engines such as Google. The search strategy included a combination of descriptors and keywords relating to clinical coordination (‘coordination of care’ or associated key terms with similar meaning), levels of care (‘primary care’, ‘secondary care’, ‘hospitalization’, ‘interface’, ‘cross-level’ or associated terms) and measurement tools (‘measure’, ‘indicator’ or associated key terms), making use of the Boolean operator ‘AND’. References from retrieved studies were also screened for possible omissions. The search was conducted in May 2011. Additional searches were conducted on the following organizations’ websites: Agency for Healthcare Research and Quality (AHRQ), World Health Organization (WHO), Pan American Health Organization (PAHO), Physician Consortium for Performance Improvement (PCPI), The Joint Commission, Agència de Qualitat i Avaluació Sanitàries de Catalunya (AQuAS), Observatori de Tendències de Serveis de Salut, the RAND corporation and the National Quality Forum (NQF). Studies in English, Spanish, Portuguese or Catalan which included one or more indicators of clinical coordination across levels of care were selected.

#### Selection and adaptation of indicators according to the different types and dimensions of clinical coordination

**Fig. 1:**
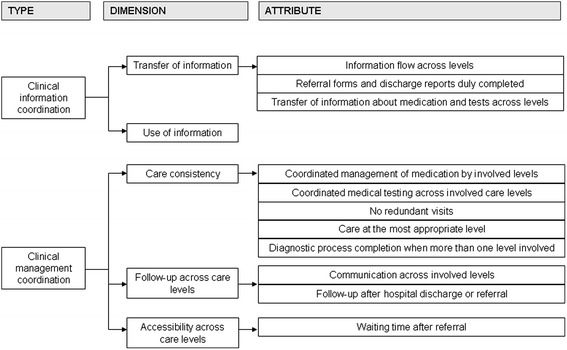
Attributes of clinical coordination across levels identified, according to the type and dimension of clinical coordination

First, the indicators included in the selected studies were analyzed to identify which attributes of clinical coordination they addressed [10]. They were subsequently grouped according to type, dimension and the attribute of clinical coordination across levels of care that they addressed (Fig. 1).

Second, three meetings took place with a team of 13 experts, who were either healthcare researchers with experience in the development of performance indicators and clinical coordination assessment or managers of healthcare services. Decisions concerning the selection and adaptation of indicators were made taking the scientific literature into account and when a consensus was reached among all participating members over three sessions of roundtable discussions. During the first meeting, it became obvious that in order to be applied, most of the indicators could not be generic but rather needed to be defined in relation to a specific disease. However, in order to gain a good grasp of the degree of coordination, a number of different diseases were included which require high levels of coordination across levels of care: diabetes mellitus type II, heart failure, chronic obstructive pulmonary disease (COPD) and breast, lung, bladder and colon cancer.

During the meetings, indicators were discussed and adapted based on the existing local clinical practice guidelines, which formed the basis on which standards of clinical coordination across levels of care were established [38–41] (for example, the guidelines allowed the team to determine when an urgent referral to secondary care is appropriate or to define the maximum acceptable time from discharge to a consultation in primary care). Each indicator was described in terms of numerator, denominator, target population, exclusion criteria, definition of terms involved, sources of data and bibliography [42].

For each indicator, the team discussed its relevance to clinical coordination across levels of care and its capacity to measure that for which it was designed (face validity). The team also discussed whether the indicator measured an aspect of care that was susceptible to being improved by services (opportunity for improvement), as well as the formulation of the indicator in such precise terms that it could be applied consistently within and between organizations, allowing for comparability (reliability). The experts identified the best sources of data to calculate each indicator (electronic medical record audit and clinical and administrative databases) and discussed its feasibility in terms of data availability and accuracy.

### 2. Test of the set of indicators

#### Design and settings

A retrospective cross-sectional study was conducted applying the set of indicators in three healthcare areas of the Catalan public healthcare system. The objectives were to evaluate the feasibility of the indicators (availability of valid, reliable and consistent data across the system) and to apply the indicators in three different healthcare areas in order to assess their usefulness in describing clinical coordination across levels of care.

Three healthcare areas were selected in order to represent the diversity of providers present in Catalonia: Baix Empordà (rural and semi-urban), the city of Girona (urban) and the Ciutat Vella district of Barcelona (urban), which all serve a population of 75,000-100,000. A single entity manages both primary and secondary care in Baix Empordà (Serveis de Salut Integrats Baix Empordà — SSIBE) and in Girona (Institut Català de la Salut — ICS). In Ciutat Vella, two public entities manage primary care (ICS and Institut de Prestacions d’Assistència Mèdica al Personal Municipal — PAMEM) and a different public entity manages secondary care (Parc Salut Mar). With regard to the coordination mechanisms used in these areas, patients served in Baix Empordà had a single electronic medical record for both care levels, whereas patients served in the other two areas had two shared but different electronic medical records for primary and secondary care. Several additional mechanisms have been implemented to improve clinical coordination across levels of care within the organizations, such as shared clinical guidelines, online consultations between primary care physicians and specialists, automated notification of primary care following hospital discharge and clinical case discussions between the two care levels.

#### Study population, data source and sample

The study population consisted of patients who had the selected conditions and who had used more than one care level, i.e. they were discharged from hospital, had received outpatient secondary care, were referred to secondary care or were newly diagnosed in primary care, depending on the indicator (Tables 1 and 2).

**Table 1:** Indicators related to clinical information coordination across levels of care

Dimension attribute		Description	Formula	Source of data	Adapted from
Information transfer					
	Information flow across levels	IT1. Percentage of hospital discharges for which a discharge report is made available to primary care within the first 24 h	- Numerator: Discharge report available in primary care within the first 24 h after hospital discharge	Discharge reports in EMRs	[22, 30, 34, 37, 49]
			- Denominator: Hospital discharges		
		IT2. Mean time to discharge report availability in primary care	- Numerator: Total hours elapsed from the time of hospital discharge to report availability in primary care	Discharge reports in EMRs	[19]
			- Denominator: Hospital discharges		
		IT3. Percentage of emergency care visits for which there is an emergency care report available in primary care within 24 h	- Numerator: Emergency care report available in primary care within 24 h of the emergency care visit	Discharge reports in EMRs	[22, 30, 34, 37, 49]
			- Denominator: Emergency care discharges		
		IT4. Mean time to emergency care report availability in primary care	- Numerator: Total hours elapsed from the emergency care visit to report availability in primary care	Discharge reports in EMRs	[19]
			- Denominator: Emergency care discharges		
	Referral forms and discharge reports duly completed	IT5. Percentage of discharge reports duly completed	- Numerator: Hospital discharge reports which contain at least four of the following items: reason for admission, additional tests performed and pending, follow-up or monitoring for the patient after discharge, list of current medications and recommendations for the patient	EMR audit	[30]
	Transfer of information on medication and tests across levels				
			- Denominator: Hospital discharge reports of patients discharged with a diagnosis of COPD, DM and/or HF		
		IT6. Percentage of emergency care reports duly completed	- Numerator: Emergency care reports which contain at least four of the following items: the reason for the emergency care visit, additional tests performed and pending (laboratory, radiology, etc.), follow-up or monitoring of the patient after the emergency care visit, list of current medications and recommendations for the patient	EMR audit	[30]
			- Denominator: Emergency care reports of patients with COPD, DM and/or HF		
		IT7. Percentage of referral forms from primary care duly completed	- Numerator: Patients diagnosed with HF, COPD and/or DM that have been referred to secondary care with a referral form that contains relevant background morbidity, current medical treatment, and the reason for the referral	EMR audit	[30]
			- Denominator: Patients diagnosed with HF, COPD and/or DM that have been referred to secondary care		

Indicators are available at: http://www.consorci.org/coneixement/cataleg-de-publicacions/80/indicadores-de-coordinacion-asistencial-entre-niveles-documento-de-trabajo

*COPD* chronic obstructive pulmonary disease, *DM* diabetes mellitus, *HF* heart failure, *EMR* electronic medical record

**Table 2:** Indicators related to clinical management coordination across levels of care

Dimension attribute		Description	Formula	Source of data	Adapted from
Care coherence					
	Coordinated medical testing across involved care levels	CC1. Percentage of secondary care visits of patients diagnosed with HF in which the specialist ordered tests that were performed in the previous six months in primary care	- Numerator: First secondary care visit of HF patients referred from primary care in which the specialist ordered a non-urgent, non-priority X-ray of the thorax, ECG or general blood test that was performed in the previous six months in primary care	Clinical and administrative databases	[50]
			- Denominator: Total first non-urgent, non-priority secondary care visits of patients referred from primary care for HF		
		CC2. Percentage of pneumology visits of patients diagnosed with COPD in which the specialist ordered a spirometry that was performed in the previous six months in primary care	- Numerator: First non-urgent, non-priority pneumology visit of COPD patients referred from primary care in which the specialist ordered a spirometry that was performed in the previous six months in primary care	Clinical and administrative databases	[50]
			- Denominator: Total first non-urgent, non-priority pneumology visits of patients referred from primary care for COPD		
	Coordinated management of medication by involved levels	CC3. Percentage of patients with DM who started insulin therapy during hospitalization and whose primary care medical record documents a follow-up within one week of discharge	- Numerator: Patients with DM who started insulin therapy during hospitalization and whose primary care medical record documents a follow-up within one week of discharge	Clinical and administrative databases	[36, 47]
			- Denominator: Patients with DM who started insulin therapy during hospitalization		
	Care at the most appropriate level	CC4. Percentage of patients with HF correctly referred from primary care to non-urgent outpatient secondary care	- Numerator: Patients diagnosed with HF and correctly referred to cardiology or internal medicine	EMR audit	[17]
			- Denominator: Patients diagnosed with HF that have been referred from primary care to cardiology or internal medicine		
		CC5. Percentage of patients with HF that have been correctly referred to emergency care from primary care	- Numerator: Patients with exacerbation of HF that have been correctly referred to emergency care from primary care	EMR audit	[17]
			- Denominator: Patients that visit emergency care for decompensated HF referred by primary care		
	Completion of diagnostic process when more than one level is involved	CC6. Percentage of patients with HF diagnosed in the past year who had an echocardiogram as part of the diagnostic process	- Numerator: Patients diagnosed with HF who had an echocardiogram as part of the diagnostic process	Clinical and administrative databases	[47]
			- Denominator: Total of patients diagnosed with HF		
Follow-up across levels					
	Communication between involved levels	FU1. Percentage of hospital discharges with contact between the hospital and primary care prior to the discharge of patients hospitalized for severe exacerbation of COPD	- Numerator: Hospital discharges with principal diagnosis related to the severe exacerbation of COPD and in which the hospital has contacted primary care prior to the discharge	Clinical and administrative databases	[21, 24, 32, 37, 44, 60]
			- Denominator: Hospital discharges with principal diagnosis related to severe exacerbation of COPD		
		FU2. Percentage of hospital discharges with contact between the hospital and primary care prior to the discharge of patients hospitalized for decompensated HF	- Numerator: Hospital discharges with principal diagnosis related to decompensated HF in which primary care has been contacted prior to discharge	Clinical and administrative databases	[21, 24, 32, 37, 44, 60]
			- Denominator: Hospital discharges with principal diagnosis related to decompensated HF		
	Follow-up visits after hospital discharge	FU3. Percentage of hospital discharges of patients admitted for exacerbation of COPD who have a consultation in primary care in less than 72 h	- Numerator: Hospital discharges with principal diagnosis related to severe exacerbation of COPD and with a consultation in primary care in less than 72 h	Clinical and administrative databases	[21, 24, 32, 37, 44, 60]
			- Denominator: Hospital discharges with principal diagnosis related to severe exacerbation of COPD		
		FU4. Percentage of hospital discharges of patients admitted for decompensated HF who have a consultation in primary care in less than 7 days	- Numerator: Hospital discharges with principal diagnosis related to decompensated HF and with a consultation in primary care in less than 7 days	Clinical and administrative databases	[21, 24, 32, 37, 44, 60]
			- Denominator: Patients discharged with principal diagnosis related to decompensated HF		
Accessibility across levels					
	Waiting time after referral	AAL1. Mean time elapsed from non-urgent, non-priority primary care referral of HF patients to cardiologist visit	- Numerator: Total days elapsed from non-urgent, non-priority, primary care referral of HF patients to cardiologist visit	Clinical and administrative databases	[61, 62]
			- Denominator: Total HF patients with non-urgent, non-priority referrals from primary care to cardiology		
		AAL2. Mean time elapsed from the referral of a patient with suspected cancer (lung, colorectal, breast, bladder and prostate) to the first specialist care visit	- Numerator: Total days elapsed from the primary care referral of a patient with suspected cancer to the first appointment with rapid diagnosis program	Clinical and administrative databases	[61, 62]
			- Denominator: Total patients referred from primary care to specialist care for suspected cancer (lung, colorectal, breast, bladder and prostate)		
		AAL3. Mean time elapsed from the referral of a patient with suspected cancer (lung, colorectal, breast, bladder and prostate) to time of cancer diagnosis	- Numerator: Total days elapsed from the primary care referral of a patient with suspected cancer to the diagnosis of cancer	Clinical and administrative databases	[61, 62]
			- Denominator: Total patients with suspected cancer (lung, colorectal, breast, bladder and prostate) first identified in primary care and with a later diagnosis of cancer		
		AAL4. Mean time elapsed from the referral of a patient with suspected cancer (lung, colorectal, breast, bladder and prostate) to the initiation of cancer treatment (surgery and/or chemotherapy and/or radiotherapy)	- Numerator: Total days elapsed from the referral from primary care of a patient with suspected cancer to the initiation of cancer treatment (surgery and/or chemotherapy and/or radiotherapy)	Clinical and administrative databases	[61, 62]
			- Denominator: Total patients diagnosed with cancer (lung, colorectal, breast, bladder and prostate) referred to secondary care from primary care who initiate treatment including surgery, chemotherapy and/or radiotherapy at the hospital to which they were referred from primary care		

Indicators are available at: http://www.consorci.org/coneixement/cataleg-de-publicacions/80/indicadores-de-coordinacion-asistencial-entre-niveles-documento-de-trabajo

*COPD* chronic obstructive pulmonary disease, *DM* diabetes mellitus, *HF* heart failure, *EMR* electronic medical record

Two sources of data were used: a) electronic medical record audit, to calculate seven indicators (five related to clinical information coordination across levels of care and two related to clinical management coordination across levels of care); b) clinical and administrative electronic databases (which differ from patients’ individual electronic medical records in the fact that they collate patient data), to calculate twelve indicators (all related to clinical management coordination).

For indicators based on electronic medical record audits, the sample size was calculated to estimate proportions, which were expected to be around 0.50; the margin of error was ±0.15 and alpha error 0.05. The sample size required was 42 patients. A simple random sample without replacement was selected from records provided by primary care centres and hospitals. For indicators based on electronic databases, all records were selected.

#### Data collection

Instructions for the data collection procedure were developed and systematically applied. Problems during data collection and analysis were recorded.

For indicators based on electronic medical record audits, data was retrieved by one researcher using standardized forms. For indicators based on databases, primary care centres and hospitals of the healthcare areas provided clinical and administrative electronic databases. Information was retrieved based on specified procedures, which had to be adapted to each information system.

#### Data analysis

In the case of dichotomous indicators, percentages were calculated, and 95 % confidence intervals were estimated when indicators were based on electronic medical record audits. Means and standard deviation were calculated for continuous indicators. Problems during data collection and analysis were discussed with the group in order to assess the applicability of the indicators and identify the main barriers to their implementation.

### Ethical considerations

The principles of confidentiality and anonymity were upheld in the researchers’ conduct, reporting, and storage of data arising from this study, in accordance with European and Spanish legislation on ethical research [43]. The study protocol was approved by the Ethical Committee for Clinical Research ‘Parc Salut Mar (2010/4124/I)’.

## Results

### 1. Development of a set of indicators to measure clinical coordination across levels of care

#### Identification of indicators: literature review

A total of 892 documents were identified: 863 from bibliographic databases, 11 from organizations’ websites and 18 from references in retrieved studies (Appendix 1). Of these documents, 862 were excluded because they did not describe nor use indicators of clinical coordination across levels of care, and 30 met the inclusion criteria, containing at least one indicator. From these documents, 52 indicators were initially identified [17, 19, 21–24, 27, 28, 30–34, 36, 37, 44–50].

#### Selection and adaptation of indicators according to the different types and dimensions of clinical coordination

**Fig. 2:**
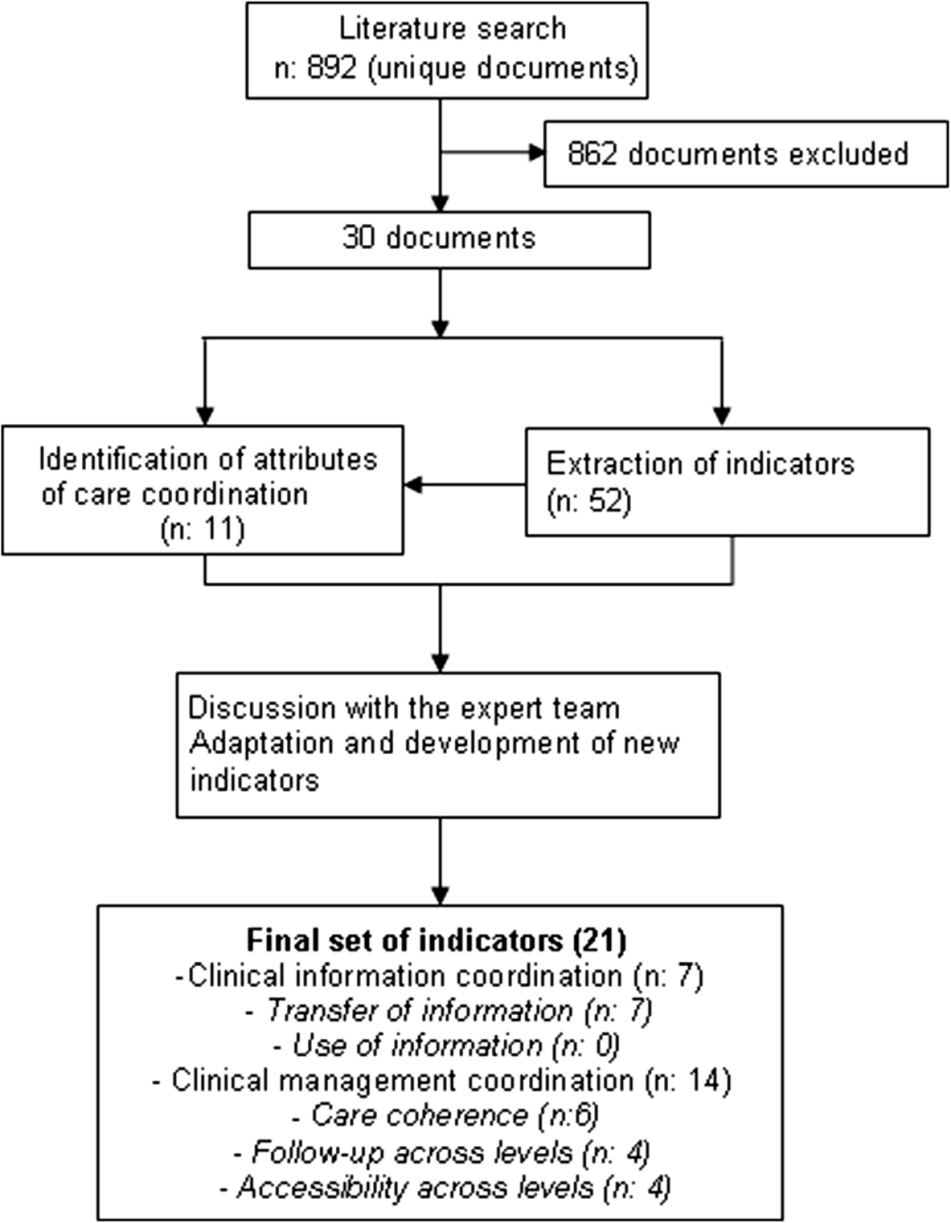
Stages in the development of the set of indicators

The 52 indicators addressed 11 different attributes of clinical coordination across levels of care (Fig. 2): 3 related to clinical information coordination and 8 related to clinical management coordination. The dimension “use of information” was not addressed by any attribute or indicator. After two meetings, an initial set of 21 indicators was drawn up (Fig. 2), which addressed 10 of the 11 identified attributes, since it was not possible to establish an unambiguous criterion which would permit the identification of redundant consultations. The remaining attributes were represented by at least 1 indicator.

The final set of indicators was as follows (Table 1 and 2):

- Clinical information coordination across levels of care: 7 indicators measure the transfer of clinical information across care levels, addressing the availability of inpatient and emergency discharge reports in primary care (four indicators) and the completeness of inpatient and emergency discharge reports and referral forms, including the transfer of information on new medication, medical tests, reasons for referral and information for patients (three indicators). No indicator addressed the dimension “use of transferred information” since it was not possible to identify or design any indicator measuring the effective use by professionals of information generated in the other care level.
- Clinical management coordination across levels of care: 6 indicators address care coherence by measuring the coordinated management of medical testing in primary and secondary care of patients with heart failure and COPD (2 indicators), the adequacy of the referral of heart failure patients from primary care to non-urgent outpatient secondary care or emergency care (2 indicators) and the completion of the diagnostic process for heart failure, which requires coordination between the two care levels (1 indicator). Four indicators measure the follow-up of patients, addressing the communication between the hospital and the primary care centre when patients with heart failure and COPD are discharged (2 indicators) and their follow-up in primary care after being discharged (2 indicators). Finally, four indicators measure accessibility across care levels, specifically the time elapsed from the primary care referral of patients with heart failure or suspected cancer to their first specialist care appointment (2 indicators) and the time elapsed from the suspicion of cancer in primary care to cancer diagnosis or initiation of treatment (2 indicators).

### 2. Test of the set of indicators

#### Clinical care information: transfer of clinical information across levels of care

In the three healthcare areas, inpatient and emergency discharge reports were immediately available in primary care, since the two care levels share electronic medical records. In general, the quality of transferred clinical information was high (i.e. the clinical information required for the transfer of patients between care levels is duly registered; for example, in referral forms: background morbidity, current medical treatment and reason for referral), especially with respect to the completeness of inpatient and emergency discharge reports, although there are notable differences between areas (57.1 % of discharge reports duly completed in Baix Empordà as opposed to 95.2 % in Girona) (Table 3). In contrast, there were low percentages of duly completed referral reports in two of the healthcare areas (11.9 % and 26.2 % of reports).

**Table 3:** Application of the set of indicators related to clinical information coordination across levels of care

Indicator			Baix Empordà	Girona	Ciutat Vella	
					ICS- Parc de Salut Mar	PAMEM- Parc de Salut Mar
IT1. Percentage of hospital discharges for which a discharge report is made available to primary care within the first 24 h		%	100 %	100 %	100 %	100 %
IT2. Mean time to discharge report availability in primary care		hours	immediate	immediate	immediate	immediate
IT3. Percentage of emergency care visits for which there is an emergency care report available in primary care within 24 h		%	100 %	100 %	100 %	100 %
IT4. Mean time to emergency care report availability in primary care		hours	immediate	immediate	immediate	immediate
IT5. Percentage of discharge reports duly completed (at least four of the five selected items)		% (95 % IC) n	57.1 % (41.5-72.7) n: 42	95.2 % (88.5 -100) n: 42	83.3 % (65.6- 91.5) n: 42	83.3 % (65.6-91.5) n: 42
	Reason for admission	% (95 % IC)	100 %	95.2 % (88.5 - 100)	97.6 % (92.7 - 100)	100 %
	Additional tests performed and pending	% (95 % IC)	95.2 % (88.5 - 100)	97.6 % (92.7 - 100)	95.2 % (88.5 - 100)	88.1 % (74.7- 96.7)
	Follow-up or monitoring of the patient after discharge	% (95 % IC)	64.3 % (49.2 - 79.0)	97.6 % (92.7 - 100)	92.9 % (84.7 - 100)	88.1 % (74.7- 96.7)
	List of current medications	% (95 % IC)	88.1 % (77.9 - 98.31)	92.9 % (84.7 - 100)	88.1 % (77.9 - 98.3)	83.3 % (65.6- 91.5)
	Recommendations for the patient	% (95 % IC)	0 %	97.6 % (92.7 - 100)	26.2 % (12.3 - 40.1)	16.7 % (6.7-31.4)
IT6. Percentage of emergency care reports duly completed (at least four of the five selected items)		% (95 % IC) n	85.4 % (74.1-96.7) n: 41	85.7 % (74.6-96.7) n: 42	86.7 % (73.8 - 100) n: 30	64.3 % (49.2-79.4) n: 42
	Reason for admission	% (95 % IC)	100 %	100 %	100 %	100 %
	Additional tests performed and pending	% (95 % IC)	92.7 % (84.4 - 100)	100 %	90 % (78.6 - 100)	88.1 % (77.9 - 98.3)
	Follow-up or monitoring of the patient after discharge	% (95 % IC)	90.2 % (80.7 - 99.7)	97.6 % (92.7 - 100)	90% (78.6 - 100)	76.2 % (62.8 - 89.6)
	List of current medications	% (95 % IC)	97.6 % (92.6 - 100)	88.1 % (74.7- 96.7)	90 % (78.6 - 100)	85.71 % (74.6 - 96.7)
	Recommendations for the patient	% (95 % IC)	19.51 % (6.8 - 32.2)	50 % (34.2 - 65.8)	43.3 % (25.5 - 62.2)	33.3 % (18.5 - 48.2)
IT7. Percentage of referral forms from primary care duly completed		% (95 % IC) n	26.2 % (12.3- 40.1) n:42	71.4 % (57.2-85.7) n: 42	11.9 % (1.7 -22.1) n: 42	88.5 % (75.3 - 100) n:26
	Background morbidity	% (95 % IC)	90.5 % (81.2 - 99.7)	95.2 % (88.5 - 100)	86.7 % (73.8 - 100)	100 %
	Current medical treatment	% (95 % IC)	30.9 % (16.4 - 45.5)	90.8 % (81.2 - 99.7)	16.7 % (4.9 - 28.4)	96.1 % (88.0 - 100)
	Reason for the referral	% (95 % IC)	90.7 % (81.2 - 99.7)	57.1 % (41.5 - 72.7)	76.2 % (62.7 - 89.6)	88.5 % (75.3 - 100)

*CI* confidence interval, *COPD* chronic obstructive pulmonary disease, *DM* diabetes mellitus, *HF* heart failure, *SD* standard deviation

#### Feasibility

All indicators were feasible in the three healthcare areas; however, in some cases the specified sample size was not reached due to an insufficient number of cases per year or due to insufficient precision in the available data to allow identification of the denominator – more than 100 records were reviewed without reaching the required sample size.

#### Clinical management coordination across levels of care

##### Care coherence

Indicators showed different degrees of test duplication (coordinated medical testing across levels of care) depending on the type of medical test performed: the highest level of duplication was observed in electrocardiograms for patients with heart failure (48 %) and the lowest was observed in spirometries for patients with COPD (2.5 %) (Table 4). In terms of care at the most appropriate care level, indicators showed high levels of adequate referral to non-urgent and emergency care. Finally, in two healthcare areas there were low percentages of patients (13.9 % and 22.7 %) who had had an echocardiogram performed in the year prior to the diagnosis of heart failure (completion of the diagnostic process).

**Table 4:** Application of the set of indicators related to clinical management coordination across levels of care

Indicator			Baix Empordà	Girona	Ciutat Vella	
					ICS- Parc de Salut Mar	ICS- Parc de Salut Mar
CC1. Percentage of secondary care visits of patients diagnosed with HF in which the specialist ordered tests that were performed in the previous six months in primary care	Duplication of radiographies	%; n	3.6 %; n:56	-	-	-
	Duplication of electrocardiograms	%; n	48.2 %; n:56	-	-	-
	Duplication of analytics	%; n	16.1 %; n:56	-	-	-
CC2. Percentage of pneumology visits of patients diagnosed with COPD in which the specialist ordered a spirometry that was performed in the previous six months in primary care		%; n	2.5 %; n:81	-	-	-
CC3. Percentage of patients with DM who started insulin therapy during hospitalization and whose primary care medical record documents a follow-up within one week of discharge		%; n	-	-	-	-
CC4. Percentage of patients with HF correctly referred from primary care to non-urgent outpatient secondary care		% (95 % IC) n	85.7 % (69.4-100) n:42	81.0 % (68.9-93.3) n:42	83.3 % (75.6-95.1) n:42	88.5 % (75.3-100) n:26
CC5. Percentage of patients with HF that have been correctly referred to emergency care from primary care		% (95 % IC) n	97.4 % (92.0-100) n:39	-	-	95.2 % (88.5-100) n:42
CC6. Percentage of patients with HF diagnosed in the past year who had an echocardiogram as part of the diagnostic process		%; n	22.7 %; n:203	13.9 %: n:216	-	-
FU1. Percentage of hospital discharges with contact between the hospital and primary care prior to the discharge of patients hospitalized for severe exacerbation of COPD		%; n	58 %; n:88	36.8 %; n:407	3.2 %; n:95	0 %; n:49
FU2. Percentage of hospital discharges with contact between the hospital and primary care prior to the discharge of patients hospitalized for decompensated HF		%; n	40.3 %; n:119	44.78 %; n:201	0 %; n:48	2.78 %; n:36
FU3. Percentage of hospital discharges of patients admitted for exacerbation of COPD who have a consultation in primary care in less than 72 h		%; n	76.7 %; n:86	52.9 %; n:240	26.0 %; n:68	53.3 %; n:45
FU4. Percentage of hospital discharges of patients admitted for decompensated HF who have a consultation in primary care in less than 7 days		%; n	70.9 %; n:110	79.6 %; n:157	55.3 %; n:38	70.6 %; n:19
AAL1. Mean time elapsed from non-urgent, non-priority primary care referral of HF patients to cardiologist visit		Mean (SD); n	28.2 (4.0); n:42	-	39.6 (5.6); n:57	100.9 (9.1); n:86
AAL2. Mean time elapsed from the referral of a patient with suspected cancer (lung, colorectal, breast, bladder and prostate) to the first specialist visit		Mean (SD); n	5.3 (0.3); n:362	-	6.5 (0.4); n:87	6.6 (1.0); n:17
AAL3. Mean time elapsed from the referral of a patient with suspected cancer (lung, colorectal, breast, bladder and prostate) to cancer diagnosis		Mean (SD); n	46.9 (9.7); n:70	-	31.4 (4.6); n:36	39.9 (8.5); n:8
AAL4. Mean time elapsed from the referral of a patient with suspected cancer (lung, colorectal, breast, bladder and prostate) to the initiation of cancer treatment (surgery and/or chemotherapy and/or radiotherapy)		Mean (SD); n	71.4 (9.2); n:64	-	48.1 (5.1); n:33	46.9 (5.8); n:8

*CI* confidence interval, *COPD* chronic obstructive pulmonary disease, *DM* diabetes mellitus, *HF* heart failure, *SD* standard deviation

#### Follow-up across care levels

In terms of communication, there were significant differences in the degree to which hospitals communicate with primary care prior to the discharge of heart failure or COPD patients (58 % and 3.2 % of COPD patient discharges in Baix Empordà and Ciutat Vella respectively). Similarly, with regard to follow-up after hospital discharge, there were marked differences between areas (follow-up of patients with COPD in primary care ranged from 26 % in Ciutat Vella to 76.7 % in Baix Empordà).

#### Accessibility across care levels

The average time waited to access secondary non-urgent care for heart failure patients referred to cardiology was higher than three weeks in all cases, with significant variations across areas. In contrast, the average time waited to access urgent care for patients with suspected cancer was lower than a week in all healthcare areas, with little variation between areas.

#### Feasibility

Five indicators were feasible in the three healthcare areas, six indicators were feasible in two healthcare areas, two indicators were feasible in only one area and lastly, one indicator was not feasible in any of the healthcare areas. Difficulties in calculating indicators were due to two types of problems. Firstly, problems related to the identification of the denominator: not possible to identify patients who had started insulin therapy (3 areas), patients referred for the first time to the secondary care level (2 areas), and patients referred to secondary care for suspected cancer (1 area). Secondly, problems related to the availability and accuracy of data needed to calculate the numerator: reason for seeking emergency care not recorded (2 areas), echocardiograms conducted in secondary care not systematically registered (1 area).

## Discussion

Clinical coordination is considered a health policy priority, as a lack of coordination can lead to poor quality of care and inefficiencies in the use of resources [5–9]. However, its measurement is still challenging [1, 14, 15], since calculating the degree of clinical coordination in its multidimensional nature requires the availability of indicators that cover the different types and dimensions of clinical coordination.

Until now, most attempts to tackle this challenge have focused on the design of indicators to measure certain outcomes which can potentially be attributed to clinical coordination [16]. However, progress must be made in the design of instruments to measure the outputs of clinical coordination in order to be able to attribute improvements in the outcomes of health care to improvements in clinical coordination [51]. With this in mind, this research constitutes a step forward by using a pre-established conceptual framework to generate a set of output indicators which address the two types of clinical coordination across levels of care (and most of their dimensions and attributes) that have been highlighted in previous studies [52]. Furthermore, in contrast with previous efforts [17, 37], the indicators presented here have been described in operative terms, thus allowing for their precise application in healthcare organizations.

With regard to clinical information coordination across levels of care, seven indicators addressing the transfer of clinical information have been created. Applying these indicators has permitted the analysis of transfer of information in the three healthcare settings, taking in both evidence of information transfer between levels and the quality of the information transferred. No previous set of indicators has allowed researchers to address these two attributes jointly in main transitions between levels of care [1, 30, 34, 53], so this is one of its most significant contributions. In addition, the result of the applicability test has proven that these indicators have the accuracy and feasibility needed to make their calculation possible in different healthcare areas. Their joint application has revealed that, although there is a flow of information between the different care levels, the quality of information varies across transitions and organizations, thus leading to the identification of specific margins of improvement in each healthcare area.

It is important to highlight, however, that clinical information coordination is not fully represented by the set of indicators, since we were unable to address the use of transferred information; i.e. we could not determine whether information was actually read and used by the receiving professional [54]. The lack of this type of measure of clinical coordination has been previously expressed in the literature [1, 54] and reflects the complexity of analyzing an activity which is not generally recorded but is considered central to clinical coordination. The fact that indicators are unable to systematically address all dimensions and attributes of clinical coordination points to the need to complement and enrich indicator results with those that can be obtained via different techniques, such as surveys or qualitative interviews with health professionals and patients.

With regard to clinical management coordination across levels of care, the systematic review led us to identify five attributes that define care coherence, two that define follow-up and one that defines accessibility across care levels. Their operationalization has resulted in a set of indicators to measure the main attributes of care coherence (such as coordinated medical testing across care levels or the provision of care at the most appropriate care level), follow-up (such as the existence of communication and follow-up after discharge) and accessibility across care levels (waiting time after referral). However, one of the eight identified attributes of care coherence, no redundant visits to primary and secondary care, is not represented by any indicator, since we were unable to establish an unambiguous criterion, either though the literature review or by expert consensus, which would permit the identification of redundant consultations.

During the first meeting, it became obvious that in order to be applied, most indicators could not be general but needed to be defined relating to a specific disease. However, in order to gain a good grasp of the degree of coordination in the area, a number of different diseases were included, which require high levels of coordination across levels of care: diabetes mellitus type II, heart failure, chronic obstructive pulmonary disease (COPD) and breast, lung, bladder and colon cancer.

The indicators to measure clinical management coordination have been adapted to several clinical conditions, due to the fact that the standards of clinical coordination upon which indicators are based need to be precise and based on what the evidence dictates, which varies according to the disease. Nevertheless, they can be adapted to other conditions as long as they have an evidence-based recommendation upon which to base the standard of clinical coordination measured by the indicator. Moreover, the use of the selected conditions (diabetes mellitus, chronic obstructive pulmonary disease, heart failure and cancer) could be considered a good strategy to identify the strengths and weakness in clinical coordination across levels of care [3, 55], since they meet the criteria to be considered adequate tracer conditions [55]: care is provided across levels and over the course of time; the care that should be provided at each care level is well defined; they are among the most prevalent diseases in the population; diagnoses are well defined; and their epidemiology is well known.

The applicability test illustrates the usefulness of these indicators in describing clinical management coordination, pointing to areas for improvement, such as the coordination of medical testing in Baix Empordà and communication with primary care after discharge in Ciutat Vella. Furthermore, as they cover the main attributes of clinical management coordination across levels, they can be used to support the design of strategies to improve clinical coordination between levels of care.

It is important to note, however, that several problems arose which made the calculation of some of these indicators difficult or impossible in some healthcare areas. The problems were related to non-registration of the variables in information systems and under-registration of information by professionals, which points to the need for further improvements in information systems and record-keeping skills before we can systematically measure certain relevant aspects related to clinical management coordination across levels of care in these healthcare areas [56–59].

The methodology adopted in this study provides guarantees in terms of reliability of the indicators, since they have been adequately defined and precisely specified so that they can be implemented consistently within and across organizations (48). This is also true in terms of face and content validity, as the indicators have been adapted or newly created on the basis of scientific evidence and expert consensus. Furthermore, the applicability test provided information regarding data availability and accuracy (feasibility), thus highlighting major and minor problems in calculating the indicators, which could be informative for future studies. Finally, the indicators have been shown to be able to identify differences between areas, even in small samples. Further research should provide evidence regarding other relevant characteristics of the indicators, such as test-retest reliability and discriminant validity.

Certain aspects should be taken into account when applying the set of indicators in other healthcare contexts. First of all, data availability and validity should be explored. Secondly, it should be determined whether the information taken from the different health information systems is linkable, since indicators are constructed upon information generated in different levels of care. Lastly, although information recorded in digital format is desirable, most indicators could be calculated from a medical record audit, so data computerization is not a prerequisite.

One limitation of this study is the possibility of a publication bias in which relevant indicators were not identified (for example, indicators that measure clinical coordination but employ different terms, indicators published in other languages or grey literature not easily accessed by standard internet search engines). Moreover, the inclusion of terms referring to certain attributes of clinical coordination, such as “follow-up” or “referral adequacy”, might have extended the range of studies obtained. However, we employed several additional strategies for the identification of studies to reduce the possibility of publication bias, such as reviewing the reference lists of eligible documents and consulting the websites of the main organizations that design indicators. Another limitation is that one dimension (the use of clinical information) and one attribute (no redundant visits to primary and secondary care) are not represented by the set of indicators, pointing to the need to enrich the results obtained by the indicators with additional information from health professionals and patients in order to attain a more accurate evaluation of the process of coordination.

## Conclusions

A set of rigorous and scientifically sound measures of clinical coordination were developed based on a literature review and discussion with experts. These indicators of clinical information and management coordination across levels of care could be employed to identify areas in which health care can be improved, as well as to measure the effect of efforts to improve clinical coordination. However, some relevant attributes of clinical coordination are not represented in the final set of indicators, which detracts from its comprehensiveness. In fact, clinical coordination is a multidisciplinary construct, and certain relevant dimensions and attributes of clinical coordination across levels of care such as the effective use of transferred information or redundant visits cannot be properly measured through indicators. Other approaches are therefore needed to obtain additional information, such as surveys or qualitative interviews. The indicators provided may also be useful for conducting comparative studies of clinical coordination across healthcare areas. Aspects such as the possibility of linking information from different health information systems, data availability and validity should be explored before proceeding to implement these indicators.

## Appendix 1

Search Strategy and number of studies retrieved in the bibliographic databases

**Table 5:** A1: medline-pubmed; 19/05/2011

	References number
1. Clinical Coordination	
"Coordinated care" OR " coordination of care" OR "integrated care" OR "shared care" OR "transitional care" OR "continuity of care"	5.573
2. Levels of care	
"Primary care" OR "family practice" OR "Generalist" OR "GP" OR "outpatient" OR "secondary care" OR "specialized" OR ‘specialist’ OR "inpatient" OR "hospitalization" OR "hospitalisation" OR "care levels" OR "interface" OR "cross-level" OR "referral" OR "communication"	655.466
3. Measurement tools	
measure OR measures OR indicator	939.670
4. (1) and (2) and (3)	466

**Table 6:** A2: Isi web of knowledge; social science citation index & science citation index 19/05/2011

	References number
1. Clinical coordination	
"Coordinated care" OR "care coordination" OR "collaborative care" OR "integrated care" OR "shared care" OR "transitional care" OR "continuity of care" OR "care continuity" OR "informational continuity" OR "managerial continuity" OR "management continuity"	5.509
2. Levels of care	
"Primary care" OR "family practice" OR "Generalist" OR "GP" OR "outpatient" OR "secondary care" OR "specialized" OR "specialised" OR ‘specialist’ OR "inpatient" OR "hospitalization" OR "hospitalisation" OR "care levels" OR "interface" OR "cross-level" OR "referral" OR "communication"	>100.000
3. Measurement tools	
measure OR measures OR indicator	>100.000
4. (1) and (2) and (3)	500
Duplicates	266
Total	234

**Table 7:** A3: ECONLIT; 19/05/2011

	References number
1. Clinical coordination	
TX (Coordinated care) OR (care coordination) OR (collaborative care) OR (integrated care) OR (shared care) OR (transitional care) OR (continuity of care) OR (care continuity) OR (informational continuity) OR (managerial continuity) OR (management continuity)	129
2. Levels of care	
TX (Primary care) OR (family practice) OR (Generalist) OR (GP) OR (outpatient) OR (secondary care) OR (specialized) OR (specialised) OR ‘specialist’ OR (inpatient) OR (hospitalization) OR (hospitalisation) OR (care levels) OR (interface) OR (cross-level) OR (referral) OR (communication)	25.224
3. Measurement tools	
TX measure OR measures OR indicator	51.184
4. (1) and (2) and (3)	4
Duplicates	3
Total	1

**Table 8:** A4: CINAHL; 19/05/2011

	References number
1. Clinical coordination	
AB (Coordinated care) OR (care coordination) OR (collaborative care) OR (integrated care) OR (shared care) OR (transitional care) OR (continuity of care) OR (care continuity) OR (informational continuity) OR (managerial continuity) OR (management continuity)	5.092
2. Levels of care	
AB (Primary care) OR (family practice) OR (Generalist) OR (GP) OR (outpatient) OR (secondary care) OR (specialized) OR (specialised) OR ‘specialist’ OR (inpatient) OR (hospitalization) OR (hospitalisation) OR (care levels) OR (interface) OR (cross-level) OR (referral) OR (communication)	74.288
3. Measurement tools	
AB measure OR measures OR indicator	104.884
4. (1) and (2) and (3)	296
Duplicates	139
Total	157

**Table 9:** A5: LILACS; 19/05/2011

	References number
((coordinación) OR (continuidad) OR (colaborativa) OR (transición) OR (compartido)) AND ((asistencial) OR (de la atención)) AND (indicador OR indicadores OR medida OR medición OR mediciones) [Palabras del resumen]	5
Duplicates	0
Total	5
